# Will Product Packaging Density Affect Pre-Purchase Recognition?

**DOI:** 10.3390/foods8080352

**Published:** 2019-08-17

**Authors:** Taebeum Ryu, Jaehyun Park

**Affiliations:** 1 Department of Industrial and Management Engineering, Hanbat National University, Daejeon 34158, Korea; 2 Department of Industrial and Management Engineering, Incheon National University, Incheon 22012, Korea

**Keywords:** size-weight illusion, confectionery box, perceived weight, preference, satisfaction

## Abstract

This study analyzed preference and satisfaction according to the weight and size of products in order to understand how the size-weight illusion (SWI) occurs in affect. Perceived weight is known to be affected not only by the weight of the object, but also by its size, color, and material. A total of 54 participants took part in the experiment. Nine kinds of cookie boxes were prepared by combining three sizes and three weight levels of confectionery products. Participants were asked to rate the perceived weight of the cookie box by the modulus method and evaluate the preference and the satisfaction of the weight by using the semantic differential (SD) scale of 11 points. The results showed that SWI occurred in terms of the perceived weight of cookies boxes like previous studies; however, SWI appeared only partially in affect. The preference and satisfaction did not increase after a certain weight, and the limits of weight were different according to the size of cookie box. These results can be referred to determine the weight and size of a product for affective design and especially utilized for the package design of cookie boxes.

## 1. Introduction

As the development of technology increases the functionality of products and the consideration of usability becomes more common, the importance of user experience (UX) in product design increases. Particularly, interest in users’ affective satisfaction is growing [1,2]. Users’ basic affects with respect to the product are unconsciously determined by the product characteristics as detected by the various senses [3]. Because basic affects influence more complex affects, such as brand image and product satisfaction, designers must be able to set a product’s sensory properties based on the target affects that they want to convey to the user. To achieve this, various research studies in the field of affective engineering are concerned with the external appearance of the product [4], visual design [5], surface material [6], characterization of operation [7] and feedback sound [8] for affective satisfaction.

The affective satisfaction experienced in a user’s sense of force, such as perceived product maneuverability and robustness, is an area that has received less attention. However, basic research on force sense has been actively conducted. The sense of strength has been studied in the field of experimental psychology. It is known that the perception of strength and weight is mainly caused by the motion command generated in the brain, and in part, the passive sensation felt in the muscles according to the exercise and touch of the skin [9]. The sense of force can be defined as the ability to discern the magnitude of force and weight with the use of voluntary muscular exertion [9]. Based on the research that analyzed the satisfaction of the exterior panel stiffness of a car, the sense of power turned out to be important in terms of affective satisfaction, and the application of the affective satisfaction technique also turned out to be highly effective [10]. Overall, research on the sense of force is still relatively insufficient compared to that on other senses.

The weight of the product has been studied in some products as an important influencing factor determining the affective satisfaction of users; however, the research on this topic continues to be lacking. The preference and affect of product weight were studied in mobile phones [11,12], writing [13], and clothing [14]. In these studies, the valence of heaviness and weight-positive affect differ according to the product. In addition, in the food industry, the influence of the weight of the packaging or container on the preference for the product has been partially recognized [15,16], but the effect on affect has not been studied actively compared to the other properties of products. Note that, of course, research on the packaging of the food itself has been actively conducted [17,18]. Additionally, some food packages may offer a healthy image, as well as a desire to buy [19,20].

It is known that the perceived weight of an object at the sensory level is influenced by the weight of the object, as well as the size, material, and color. For instance, in the case of objects of the same weight, but different sizes, we perceive heavier objects to have smaller sizes [9]. This is explained by the fact that our brain assigns a smaller amount of muscle force than the actual force required to lift the object. On the contrary, in case of a large object, the brain assigns more force than the force required to lift the object, which leads to a perception of lesser weight. The same principle applies to objects of the same weight, but of different materials, which means that wooden objects are heavier than iron objects. Additionally, for objects of the same weight, but different colors, we feel that light objects are heavier than dark ones. The demographic characteristics, such as gender and age, as well as haptic features of objects can also affect perceived weight and consumption [21,22].

However, existing studies on the affect of product weight neglected product size, which are closely related to weight and considered in the force sense studies. Existing studies on the weight of products, such as cell phones, clothing, and writing instruments do not suggest how the interaction between weight and size influences users’ affects. In addition, studies on the packaging and container weight of food products also focused on the visual perceived weight based on the position of the product image on the packaging paper or the weight of the container, but there was no consideration of the size. Generally, the weight of a product is proportional to its size, so a product design considering affective satisfaction requires setting the size together with its weight. Research on affect that considers the weight and size of a product can show preference weight for various size, that is, the preferred density of the product.

Therefore, this study seeks users’ satisfaction according to product weight and size and suggests preference weight according to product size. To meet the study objective, cookies were selected, and the satisfaction with and preference for cookies with various weights and sizes were evaluated. The selected cookie products are sold in various weights and sizes in Korea and were suitable for this study. The cookie was packed in a paper box, and it was easy to change the weight of the product. This study is based on the assumption that user satisfaction would increase based on the weight of the cookie product, but it did not increase when it was excessively heavy. In other words, this study predicted that there would be a preferred weight limit depending on the size of the cooked product, and propose it. The existing force sense studies mentioned above revealed the illusion of perceived weight according to weight and size in the sensory dimension, but this study can express affective change of product satisfaction according to weight and size.

## 2. Literature Review

Product weight has been studied for some products as an important design factor affecting users’ affective satisfaction, and valence of heaviness has been reported differently depending on the product. For mobile phones, weight is a major consideration that affects purchase decisions, along with size [23]. For example, the weight of the external attributes of the mobile phone has the greatest influence on the luxuriousness [11]. Also, as the weight of a mobile phone increases, user preference decreases, but reliability and robustness increase [12]. In the case of the tool, the heaviest weight among 6.8, 13.9, and 21.4 g weights turned out to have the highest scores on writing and comfort [13], while weight turned out to have a negative effect on affect and preference of fabric [14]. The effects of product characteristics on affective satisfaction are determined by the user’s affective state [24] and the fit of product use [25,26]. Consequently, the direction of the effect of product weight on product preference depends on the utility of the product [16]. Users generally prefer heavy items when the quantity of products is important, such as confectionery products and do not prefer heaviness when portability becomes important, such as mobile phones.

In the sensory dimension, research has been conducted on the relationship between perceived weight and vision. However, in the cognitive and affective dimension, the perceived weight has been studied inadequately despite its importance. Our sensory experience is often associated with concept recognition and is a necessary part of conceptual cognitive activity [27,28,29,30]. For example, in the sense of space, the center is important, the periphery is ancillary, the upward direction is increasing, the downward direction is decreasing, and the proximity of the distance is related to the familiar and falling is the unfamiliar concept [31]. Perceived weight is metaphorically related to the concept of importance, authenticity, and excellence [32,33]. The perceived weight in product families, such as perfume and wine bottles, influences quality and price [34]. In a social behavioral context, the weight of the clipboard on which the application was placed influences the applicant’s impression [32]. Candidates whose applications were placed on heavy clipboards were found to be more important and better rated. However, the sense of power, such as perceived weight, continues to be an important factor in our personal and social daily life, but cognition and affect in various situations has not been sufficiently studied [35].

The sensibility of a product originating from one sensation is also influenced by other senses, and attempts to grasp this from a multisensory aspect are being actively made. The relationship between product sensibility determined by one sensation and other senses is called crossmodal correspondence. For example, the flavor intensity of yogurt is affected by the visual color and shape of the wrapping paper [36]. In addition, fabric swatches having lemon or lavender fragrances appear tactually smoother than those having animal fragrances [37]. The crisp of potato chips increases when the intensity, and frequency of the food crunching sound, is high [38]. This crossmodal correspondence study can present a combination of various sensory information to transmit the target sensibility more.

The crossmodal correspondence study of the food industry has partially examined the effect of the weight of packaging or containers on the preference of the product, but the research on this topic remains insufficient. In the food industry, interest in the crossmodal correspondence of packaging or containers to content has increased, and its shape, color, texture, smell, and sound affect not only user interaction [39], but also taste experience and product evaluation [40,41,42]. However, the weight is relatively neglected. Few studies on this have been conducted as follows. Yogurt preference and the expected price of the heavy container turned out to be high [15], while perceived weight and preference seemed to be high when the product image was located at the bottom right [16]. In addition, non-overpackaged products could provide customers with environmental conscious [43].

## 3. Method

### 3.1. Participants

In this study, 61 healthy university students (41 males and 20 females) participated voluntarily. In order to estimate the perceived weight of the box in this study, a magnitude estimation ability test was performed using line length and number. Among the participants, 54 (38 males and 16 females) passed the test. Note that approximately 3–6% of the population did not pass the size estimation ability test [44]. Table 1 summarizes information about the body (height and weight) and product use (frequency of cookie purchase, intake, and preference type) related to participants who passed the size estimation capability. Participants mostly preferred cookies in confectionery products, most of them enjoyed cookies at least once a week, consumed more than half at the time of ingestion, and did not consider health when eating cookies. This research complied with the tenets of the Declaration of Helsinki and was approved by the Hanbat National University’s Institutional Review Board (P01-201709-22-001).

### 3.2. Selection of Confectionery Products

In this study, we selected cookies that were expected to have high preference and satisfaction according to the weight of the products. Confectionery products related to health were excluded from the study, assuming that weight and preference were not related or negatively correlated. The experimental cookie products were sold in at least three sizes as popular products in the Korean market, and packaged in paper boxes, with a large weight range, that were easy to handle. The small box of this product has a volume of 535.44 cm^3^ (13.8 × 9.7 × 4 cm), a medium box of 1035.72 cm^3^ (volume 18.3 × 13.7 × 4 cm) and a large box of 2067 cm^3^ (26.5 × 19.5 × 4 cm). These boxes weighed 100, 198, and 385 g, respectively.

In this experiment, there are three types of weights (100, 198, and 385 g), and three sizes (small, medium, and large). The combination of weights and sizes produced nine pairs (Table 2). In the modulus method mentioned below, the reference weight is 198 g. For this reason, the numbering was started from a box weighing 198 g.

### 3.3. Procedure

First, the magnitude estimation ability of participants was confirmed by a preliminary experiment involving estimating the number as a line length and the line length as a number [45]. Estimation ability was evaluated with regard to whether the 95% confidence interval of the regression slope after the logarithmic conversion of the line length and number included 1 [44]. Only the participants who passed this size estimation ability test evaluated the perceived weight and affect of the experiment.

Participants who had passed the preliminary experiment were informed about the purpose of the experiment, the method, and the evaluation items, and evaluated the perceived weight of the confection box, the preference, and the satisfaction with the weight of the confection box. Experiments were carried out with active lifting, in which, similar to the experiment by [46], the user felt perceived weight by placing his or her arm in one hand. This method can be regarded to be highly sensitive to perceived weight evaluation [9]. In order to make tactile and visual sense of the size information affecting the size-weight illusion (SWI), there was no limit to the hand movements required to put the box in one hand similar to the previous experiment [46]. Therefore, participants used both visual and tactile information to feel the weight of the cookie box and to evaluate sensory perceived weight and subjective sensitivity.

In this experiment, the perceived weight of the confection box was evaluated by setting the perceived weight value of the reference box according to the modulus method [47]. This method can be used when the difference between the reference stimulus and the evaluation stimulus is not large [45], and has been used in previous studies [21,46] with sizes (e.g., 140, 350, and 904 g) similar to the box sizes of this experiment. Analysis of this method is simple compared to the modified modulus, in which the participant assigns the desired numerical value to the reference stimulus and assigns the numerical value to the remaining experimental stimulus based on this, and the free modulus in which the experimental participant freely assigns the numerical value to the experimental stimulus without the reference stimulus. In this experiment, the reference stimulus was selected as a medium 198 g box among the nine boxes owing to its size and weight. The perceived weight of this box was set to 100, and the participant evaluated the perceived weight of the test boxes relative to this. The criterion box was prepared separately from the evaluation boxes and was placed next to the participant to be used before the evaluation. The perceived weight size evaluation item presented in the evaluation sheet is “If the reference box weight is 100, indicate the weight of the given box.”

The preference and satisfaction for the weight of cookies were measured using the 11-point semantic differential (SD) scale. The preference was measured by using 5 points for “no more good” and −5 points for “no more dislike” and using that to the decimal point. Satisfaction was also measured in the same manner as “no more satisfaction” with 5 points and “no further dissatisfaction” with −5 points.

Box ordering was balanced by the 9-order Latin Square method to offset the carry-over effect. If the number of stimuli is odd, then we need to use at least 18 experimental participants since we need to use both random tables generated by this method. The study involved 54 participants, which was thrice the number of participants. The participant evaluated the perceived weight of each test box once, and the experiment took about 40 min. After the experiment, a predetermined fee was given to each participant.

### 3.4. Analysis Method

This study first analyzed whether the perceived weight, preference, and satisfaction of cookies differed according to participants’ physical characteristics and experience of product use. For this, mixed-factor design analysis of variance (ANOVA) was used with the weight and size of the confectionery box as within-subject factors and the individual factors of participants as a between-subject factor. The perceived weight measured by the modulus method of this study was used for ANOVA without logarithmic transformation [45], and preference and satisfaction were the same.

The study aimed to investigate the effect of weight and size on the perceived weight, preference, and satisfaction of cookies. When the weight and size of confectionery box had a significant effect, we attempted to analyze the interaction effect by simple effect test [48].

In addition, this study attempted to develop a regression model to estimate the perceived weight in the weight and size of cookies. The perceived weight model was derived to confirm whether the results obtained in previous SWI studies are also present in this study. The relationship between perceived weight, size, and weight follows the power function of [49], so regression analysis was performed on their logarithmic transformations.

Finally, we attempted to grasp the relationship between the density of confectionery, preference, and satisfaction in order to present the preferred limit weight according to the size of the confectionery box. For this purpose, the density of the cookie box was converted into logarithm, and the preference and satisfaction were not converted. This study attempted to suggest a preference and satisfaction regression model according to the density of cookies.

## 4. Results

### 4.1. Weight and Size of Confectionery Box

The results of the repeated measures ANOVA to determine the effect of weight and size on the perceived weight, preference, and satisfaction were significant (Table 3). The repeated measures ANOVA, including weight and size, was used for analysis. The weight of the confectionery box significantly influenced perceived weight (F (2, 106) = 234.99, *p* < 0.001), preference (F (2, 106) = 45.46, *p* < 0.001), and satisfaction (F (2, 106) = 74.23, *p* < 0.001), and the size of confectionery box also influenced perceived weight (F (2, 106) = 82.02, *p* < 0.001), preference (F (2, 106) = 33.68, *p* < 0.001), and satisfaction (F (2, 106) = 56.52, *p* < 0.001) respectively. The interaction between weight and size was also significant.

### 4.2. Perceived Weight according to Weight and Size

**Figure 1:**
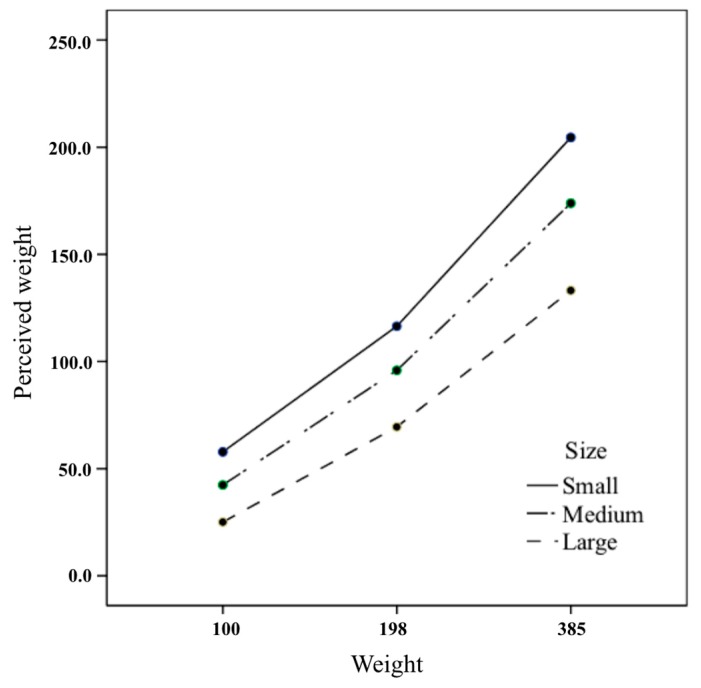
Interaction effects of size and weight of cookie box on perceived weight.

As indicated in Figure 1, the perceived weight of small boxes tended to largely increase with the increase in weight. The scale of the y-axis is based on the 198 g = 100, mentioned above. The simple effect test was used to compare multiple perceived weights among sizes at different weight levels. The least significant difference (LSD) analysis showed that the perceived weights of all sizes were different at each weight level, as shown in Table 4 (*p* < 0.001). At the same weight level, the perceived weight of the small box was significantly higher than the medium and the large boxes, and the perceived weight of the medium box was also significantly higher than that of the large box. That is, the smaller the size, the higher the perceived weight, and the tendency intensified as the weight increased.

The perceived weight between the weights at different size levels was different (Table 5), and the perceived weight among all weights at the same size level was also different (*p* < 0.001). Perceived weight of 385 g at one size level was significantly higher than 198 g and 100 g, and perceived weight at 198 g was higher than 100 g. In other words, the larger the weight, the higher the perceived weight, and this tendency intensified as the box size decreased.

The interaction between weight and size showed a tendency towards heaviness in the small box as the weight increased, but it was not confirmed by the regression analysis of the perceived weight according to the box size at each weight level. The perceived weight and the physical size exhibited a power function relationship, and regression analysis is performed after converting them to the natural logarithm. However, since the size of the box at each weight level was only three, the size coefficient of the regression equation was not significant. Therefore, this study could not confirm whether the effect of the size on the perceived weight varied depending on the weight. However, one of the related study [46], which has nine levels of size, has already proved that the strength of the SWI that does not depend on the weight. In the previous study, the regression coefficients for size variables of the regression equation explaining perceived weight in object size were not significantly different by weight.

However, the SWI of this study was confirmed to be the same as the previous study [46]. The regression equation of perceived weight, including both size and weight, as shown in Equation (1); and the perceived weight regression equation of this study is shown in Equation (2). The 95% confidence interval of regression weight and size regression coefficients in this study included the regression coefficients of the previous study. The intercept coefficient of the existing perceived weight regression equation was different from that of the present study. This is because the previous study set the perceived weight of 100 at 350 g, while 198 g was set at the perceived weight of 100 in this study. The adjusted-R2 of the regression model of this study was 0.91 and Durbin-Watson was 2.13. The variance inflation factor (VIF) of both variables was 1.0.

log(perceived weight) = −0.09 + 1.25 × log(weight) − 0.35 × log(size)(1)

log(perceived weight) = 0.79 + 1.08 × log(weight) − 0.44 × log(size)(2)

### 4.3. Preference according to Weight and Size

**Figure 2:**
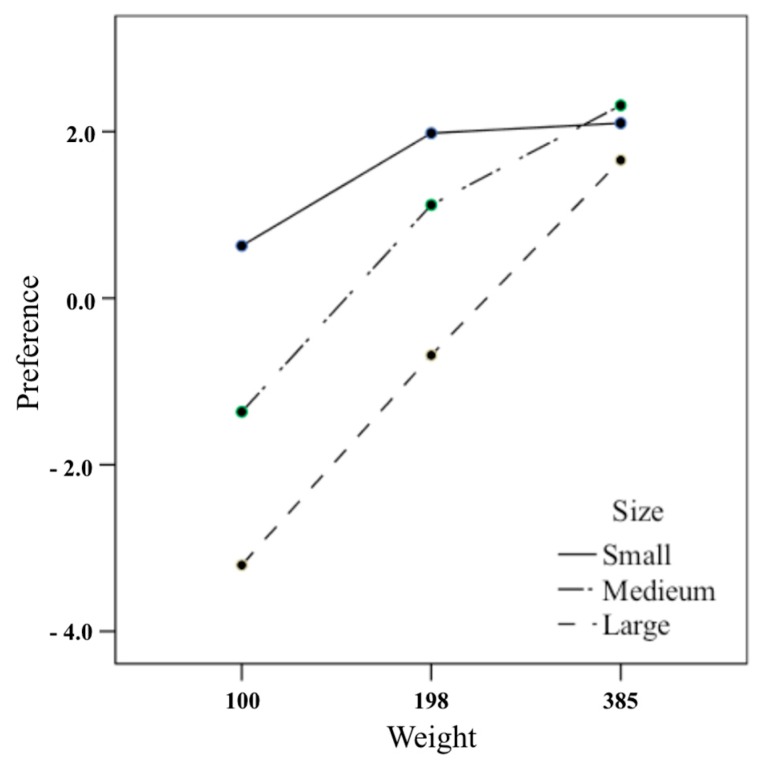
Interaction effects of size and weight of cookie box on preference.

As indicated in Figure 2, the preference of confectionery boxes increases as the weight increases in the large box, but not in the small box. In the large box, the preference of 385 g weight was significantly higher than those of 198 g and 100 g, and the preference of 198 g was significantly higher than that of 100 g (Table 6). The same results as the large box were obtained for the medium box as well, but the difference between 198 g and 385 g was not as large as the large box. On the other hand, the preference for a weight of 198 g in a small box was significantly higher than that of 100 g, but the preference for a weight of 385 g was not statistically different from that of 198 g. These results show that there was no infinite preference increase with increasing weight in one cookie box size as assumed by the study, and there was a preferred limit weight. As a result of comparing the preference according to the size of confectionery box at each weight, the preference of the box size at 100 g and 198 g significantly increased when the box size was smaller, but the preferences for the three size boxes at 385 g were all the same (Table 7).

### 4.4. Satisfaction according to Weight and Size

**Figure 3:**
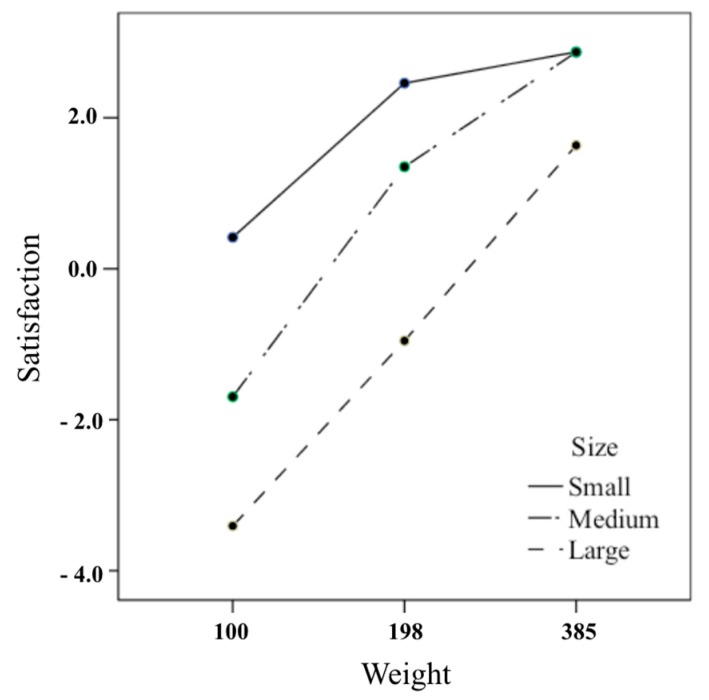
Interaction effects of size and weight of cookie box on satisfaction.

The interaction between weight and size with respect to satisfaction was similar to preference (Figure 3). In other words, the larger the weight of the large box and the medium box, the higher was the statistical significance of satisfaction. For the small box, the satisfaction of 198 g was significantly higher than that of 100 g, but the satisfaction of 385 g was not significantly larger than that of 198 g (Table 8). As a result of comparing the degree of satisfaction with the box size at each weight, the smaller the size at 100 g and 198 g, the higher was the satisfaction. The satisfaction level of the medium box was significantly higher than that of the large box at 385 g, but the difference of satisfaction between the small and medium box was not significant (Table 9). This result supports the hypothesis that satisfaction does not increase infinitely as the weight increases, but increases the marginal satisfaction weight similar to preference.

### 4.5. Density of Confectionery Box

**Figure 4:**
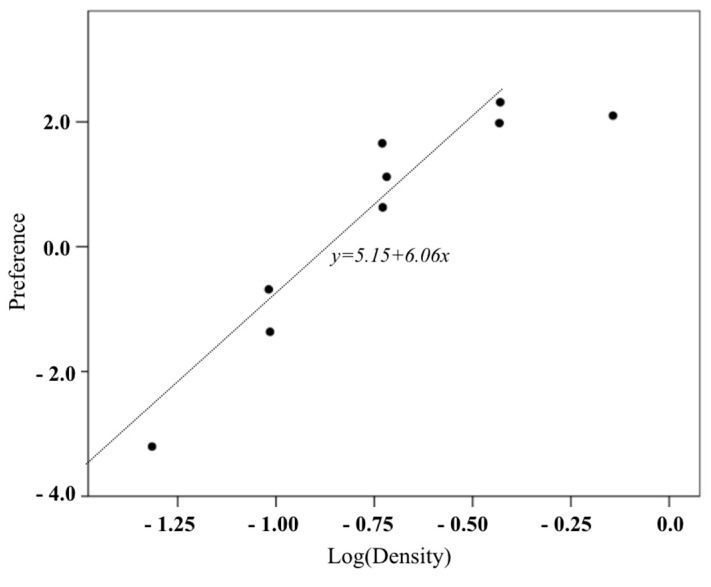
Scatter plot between preference and cookie box density.

**Figure 5:**
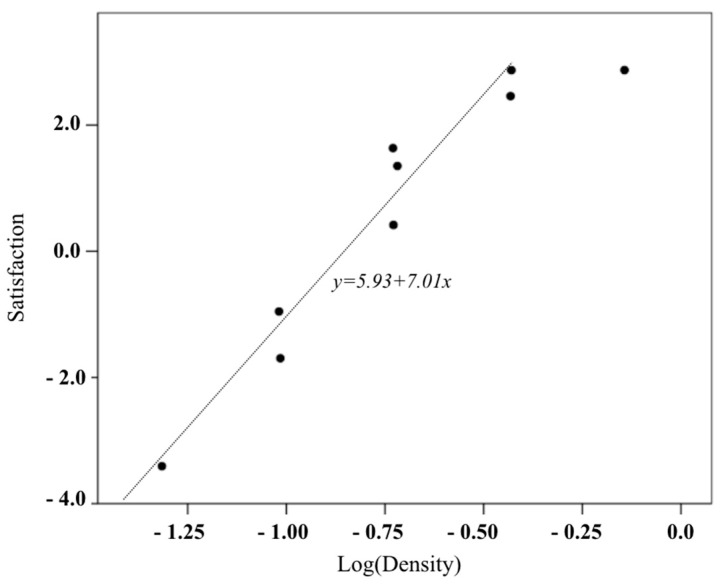
Scatter plot between satisfaction and cookie box density.

In this study, the relationship between the confectionery box density and the average preference and satisfaction of participants is shown as a scatter plot in Figure 4 and Figure 5. Note that the density was logarithmically transformed. Both satisfaction and preference linearly increased to 0.37 g/cm^3^ (logarithmic conversion value = −0.43), but no longer increased at the highest density (0.72 g/cm^3^) (logarithmic conversion value = −0.14). Table 10 shows the results of the regression analysis of the interval of density where the preference and satisfaction and the logarithmic conversion density have a linear relationship. The regression equation of preference is shown in Equation (3), and the regression equation of satisfaction is, as shown in Equation (4). Their coefficient of determination was above 0.9, and the independence of residuals and the assumption of equidistribution were also confirmed. This result implies that the weight of a certain cookie box size is determined by using the density 0.37 g/cm^3^ proposed in this study, and that satisfaction and preference were high.

(Preference) = 5.15 + 6.06 × log(Density)(3)

(Satisfaction) = 5.93 + log(Density)(4)

## 5. A Case Study for Verification of Marginal Density

A verification experiment was additionally conducted to confirm if the proposed satisfaction and preference marginal densities were maintained in different size and weight combinations. In the previous experiment, all three sizes (i.e., large, medium, and small) were set to weights of 100, 198, and 385 g. Small boxes consisted of only commercial weights (100 g) and larger weights (198 and 385 g), while the large box had only a commercial weight (385 g) and smaller weights (100 and 198 g). The experiment results revealed that the preference and satisfaction level of 198 g, which is one step higher than the sales weight (100 g), increased significantly in the case of the small box, but the preference and satisfaction level of 385 g, which is larger than this, did not increase any more. In the medium case, the preference and satisfaction level of 385 g, which is one step higher than the sales weight (198 g), was significantly higher. Whether these larger weight preferences and satisfaction would increase no further was not confirmed as there were no corresponding weight conditions. Additionally, in the case of the large box, the preference and satisfaction of the sales weight (385 g) were significantly higher than that of the smaller weight (100 and 198 g), but it was not confirmed whether the preference and satisfaction tendency of the weights were larger than the sales weight. Therefore, this additional case study verifies the proposed limit density, including the weight conditions smaller than the sales weight in case of the small box, weight conditions larger than sales weight in case of big box, and weight conditions extended from full weight range in case of the medium box.

### 5.1. Design of Experiment

In this test, 36 healthy university students (24 male and 12 female) participated voluntarily. They did not participate in the previous experiment and passed all of the line length size estimation ability tests. In this study, the weight condition of each size to be included in the verification experiment was selected based on the cookie densities of the previous experiment. The density of the confectionery box size and weight combination in the previous experiment is shown in Table 11. The weight of the cookie box was calculated considering the box volume and five densities. The weight not included in the previous experiment for each box size was used for the verification experiment (gray part of Table 11). Note that gray cells in Table 11 are included in the validation experiment, while white cells were included in the previous experiment. In the case of small boxes, 27 and 53 g were selected as the weight of the verification experiment, and the commercial weight of 100 g was also included. In the case of the medium box, 53 and 755 g were selected as the weight of the verification experiment, and the commercial sales weight of 198 g was also included. For the large box, 755 and 1480 g were selected as the weight of the verification experiment, and the commercial sales weight was also included. The selected nine condition boxes were prepared by removing the contents or using the weights similar to the previous experiment.

### 5.2. Experimental Procedure

The procedure of this verification experiment was the same as the previous experiment. Participants were informed about the purpose of the experiment, method, and evaluation items, and were given a confection box according to the randomized box number. The perceived weight, preference, and satisfaction were evaluated by the same procedure as the previous experiment. The perceived weight was evaluated according to the modulus method, and the preference and satisfaction were evaluated according to the 11-point SD scale.

### 5.3. Results

#### 5.3.1. Perceived Weight

**Figure 6:**
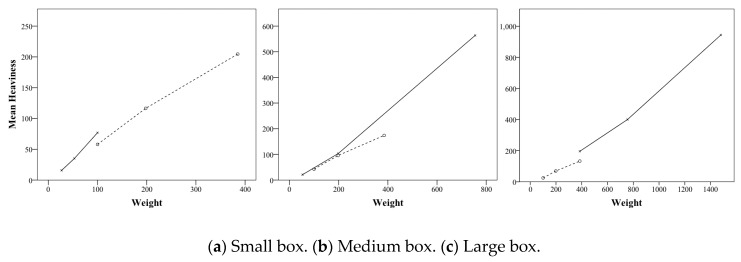
Mean perceived weight of weights for each box size (the dashed line is the first experiment, and the solid line is the second experiment).

In this test, the perceived weight tends to increase as the weight increases in all three box sizes similar to the previous experiment. Figure 6 shows the perceived weight according to the weight of the small box, medium box, and large box. It was confirmed that the perceived weight increases as the weight increases in the small weight range (27 and 53 g) added with the small box weight of the second experiment and in the large weight range (755 and 1480 g) added with the large box weight. The same results were obtained in the weight range (53 and 755 g) added by the medium box weight of the second experiment. In Figure 6, although the first and second experimental perceived weights did not match at 100 g of the small box, 198 g of the medium box, and 385 g of the large box, the perceived weight in the entire weight range increases steadily with weight.

#### 5.3.2. Preference

**Figure 7:**
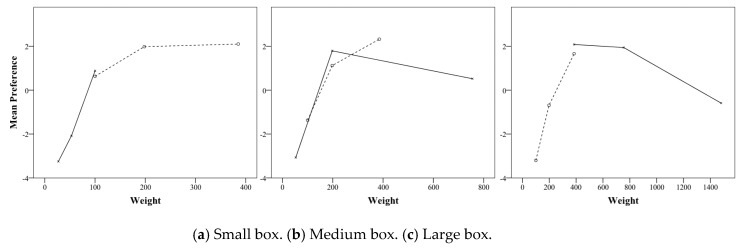
Mean preference of weights for each box size (the dashed line is the first experiment, and the solid line is the second experiment).

The results of the first experiment that preference marginal weight did not continuously increase with increasing weight were confirmed in the medium and large boxes of this verification experiment. In the first experiment, the presence of the preferred limit weight was confirmed only in the small box, but in the second verification experiment, the limit weight also appeared in the medium and large boxes. Figure 7 shows the preference according to the weight of the small box, medium box and large box, as the dotted line in the first experiment, and the solid line in the second experiment as the verification experiment. In the medium box of the first experiment, the preference of 385 g, which is one step higher than the sales weight (198 g), was significantly higher (dashed line in Figure 7b). However, in the validation experiment, the preference of 755 g, which is two steps heavier than the sales weight of the medium box, was rather smaller than the sales weight (solid line in Figure 7b). Additionally, in the large box of verification experiments, the preference of 755 g, which is one step heavier than the sales weight (385 g), did not increase anymore, and the preference of 1480 g, which is two steps heavier, was rather small (solid line in Figure 7c).

In addition, the results of the first experiment showed that the preference decreased sharply as the box weight was lower than the sales weight of the box. The same result was confirmed in the verification experiment. The preference for smaller weights (27 and 53 g) in the small boxes of the verification experiment was sharply lower than the sales weight, and the preference for weight (54 g), which is two steps less than the sales weight in the medium box, was smaller than the sales weight (198 g). This was lower than the preference score of 100 g, which was one step lower than the medium box sales weight in the first experiment.

#### 5.3.3. Satisfaction

**Figure 8:**
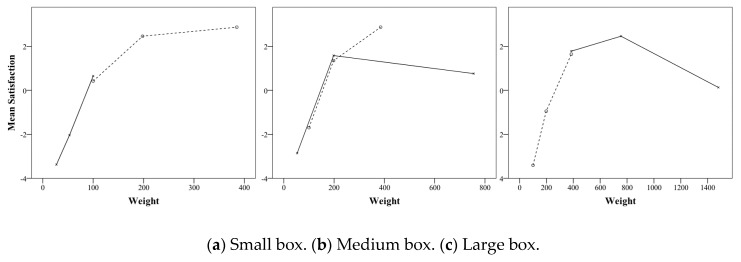
Mean satisfaction of weights for each box size (the dashed line is the first experiment, and the solid line is the second experiment).

In the first experiment, satisfaction did not continuously increase with increasing weight, but satisfaction limit weight was also confirmed in the medium box and large box of this test. The existence of the satisfactory marginal weight was confirmed only in the small box in the first experiment, but in the second verification experiment, the marginal weight also appeared in the medium box and the large box. Figure 8 shows the satisfaction with the weight of the small box, medium box and large box, with the dotted line in the first experiment, and the solid line in the second experiment. In the medium box of the first experiment, the satisfaction level of 385 g, which is one step higher than the sales weight (198 g), was significantly higher (dashed line in Figure 8b), but in the verification experiment, the 755 g satisfaction, which was included in the two-step heavy weight of the medium box’s sales weight, was rather smaller than the sales weight (solid line in Figure 8b). Additionally, in the large box of the verification experiment, 755 g of one step heavier than the sales weight (385 g) was higher than the sales weight, but satisfaction with the two-stage heavy 1480 g was rather small (solid line in Figure 8c).

The results of the first experiment and the verification experiment showed that the lighter the box was, the less was the drastic reduction in satisfaction. The satisfaction with small weights (27 and 53 g) smaller than the sales weight in the small box of the verification experiment was sharply lower than the sales weight (Figure 8a solid line). In the medium box, the satisfaction with two steps less than the sales weight (54 g) was less than the sales weight (198 g) (Figure 8b solid line), which was lower than the medium box sales weight in the first experiment, lower than the satisfaction score of 100 g.

#### 5.3.4. Density of Confectionery Box

**Figure 9:**
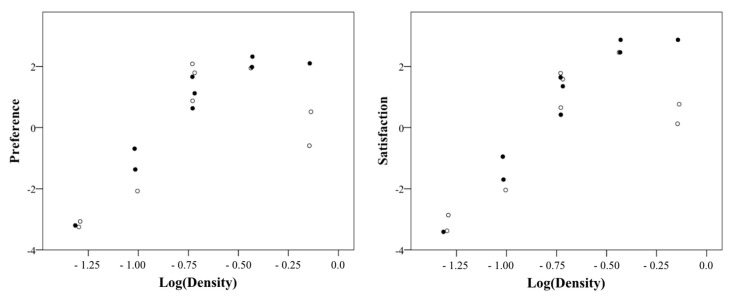
Scatter plot of preference and satisfaction with cookie box density (the black dot is the first experiment, and the white dot is the second experiment).

This study adds verification test data to balance the data of each density level in the scatter plot among the box density, preference, and satisfaction of the first experiment (Figure 9). At density levels other than the largest density, the black point of the first experiment and the white point of the verification experiment were close to each other, confirming that the relationship among density, preference, and satisfaction of the first experiment was maintained in the verification experiment. In both experiments, both satisfaction and preference increased linearly to 0.37 g/cm^3^ (logarithmic transformed value = −0.43), but no longer increased or decreased at the highest density (0.72 g/cm^3^) (logarithmic transformed value = −0.14). Table 12 presents the results of the regression analysis of the density interval in which the logarithmic transformed density has a linear relationship with the preference and satisfaction in the two experimental data. Both regression models showed high explanatory power, residual independence, and equispaced distribution. Therefore, the preference and satisfaction limit density were 0.37 g/cm^3^ in the verification test.

## 6. Discussion

Existing experimental psychology studies have studied perceived weight according to the weight and size of an object, but this study has significance in studying affect (i.e., preference and satisfaction) according to the object size. Existing SWI studies have analyzed the illusion of perceived weight according to the weight and size of an object as a field of force sense, and found that perceived weight increases as the object size decreases at the same weight [9,21,46]. In these studies, perceived weight, preference, and satisfaction were examined similarly. Note that attempting to identify SWI characteristics in terms of preference and satisfaction is a major contribution of this study. The perceived weight of the confectionery box was the same as the SWI result of the previous studies, and preference and satisfaction increased with the same weight, but it did not appear any more than the certain limit weight. In the study revealed that the perceived weight, which is the sensory dimension, has an infinite linear relationship with the weight and is inversely proportional to the size, but that the affective dimension, preference, and satisfaction have this relationship limitedly. This means that the perceived weight, which is the sensory dimension, is mainly determined by external stimuli, while the affective dimension, preference, and satisfaction are determined by internal factors, such as cognitive experience, as well as external stimuli.

This study has the same result as the SWI phenomenon found in previous studies in the perceived weight analysis of confectionery boxes, implying that this study appropriately used the experimental and analytical methods of previous studies. At the 100 g weight of the first experiment, the small box showed significantly greater perceived weight than the medium box and the large box, and the medium box showed significantly greater perceived weight than the large box, which was the same at 198 g and 385 g. Thus, the perceived weight of the box in this study increased significantly as the size decreased. In addition, the coefficients of the regression model that estimated the perceived weight of the confection box in terms of weight and size were statistically similar to those of [46]. This is explained by the fact that this study used the modulus method for estimating the perceived weight of confection boxes and the logarithmic transform for perceived weight regression analysis as in previous studies [21,46].

This study has experimentally proved that there is a limit of weight that is preferred and satisfactory in a certain size of a cookie box. The study is based on the assumption that the preference and satisfaction of confectionery products do not increase continuously according to their weight, but have limitations, unlike perceived weight, which increases the proportionally with weight. As a result of the first experiment on nine kinds of confectionery boxes of three weights (i.e., 100, 198, and 385 g) and three sizes (i.e., large, medium, and small), the preference and satisfaction of one size of confectionery tend not to increase more than the specific weight. In the small box, the preference and satisfaction of 198 g weight increased significantly compared to the sales weight of 100 g, but the preference and satisfaction of the weight of 385 g did not exceed the weight of 198 g. In the medium box, the preference and satisfaction of the weight of 385 g compared to the sales weight of 198 g demonstrated a significant increase. The degree of increase was similar to the satisfaction and preference of 198 g weight compared to the 100 g in the small box. (Figure 2 and Figure 3). These results suggest that weighing one step higher than the weight of the confectionery box sold on the market is preferred, but the preference for a two-step weight no longer increased.

The validation experiment of this study confirmed the presence of preference and satisfaction limit weight. In the limit weight results of the first experiment, the preference and satisfaction of the two-step weight (755 g, 0.72g/cm^3^) of the medium box were expected to be no longer greater than the commercial sales weight. In the case of large boxes, the preference and satisfaction level of 755 g (0.37g/cm^3^) is higher than the sales weight (385 g), but the preference and satisfaction of the two-step weight (1480 g, 0.72g/cm^3^) were not expected to increase any more. As a result of the verification, the preference of 775 g that is two steps higher than the medium box sales weight (198 g) and satisfaction decreased rather than the 198 g. Additionally, the satisfaction of 755 g, which is higher than sales weight (385 g) in the large box, was higher than 385 g, but the preference was similar, and the preference and satisfaction level of 1480 g were lower than 385 g. Therefore, the verification test of this study fully proved the prediction based on the existence of the limit weight of the first experiment.

On the other hand, the first experiment of this study shows that satisfaction and preference decrease sharply when the weight of the cookie box is smaller than the market sale weight. In the medium box of the first experiment, the preference and satisfaction level of 100 g, which is one step lower than the sales weight (198 g), decreased sharply, and the preference and satisfaction of 198 g, which is one step lower than the sales weight (385 g) also fell sharply. In addition, the preference and satisfaction level of 100 g, which is two steps smaller than the sales weight, in the large box sharply decreased than 198 g. These results indicate that as the weight of the box of confectionery was lower than the weight sold on the market, the preference, and the satisfaction reduced sharply. Due to this tendency, the preference and satisfaction level of 53 g, which is two orders of magnitude smaller than the sales weight of the medium box (198 g), is expected to drop sharply from 100 g and the preference and satisfaction of 53 g and 27 g, which are smaller than the sales weight of small boxes (100 g), are expected to decrease sharply. This prediction was proved in the verification experiment of this study. The preference and satisfaction level of 53 g, which is two steps smaller than the sales weight of the medium box (198 g), sharply decreased from 100 g, which is one step smaller than the sales weight. Additionally, the preference and satisfaction level of 53 g, which is one step lower than the sales weight of the small box (100 g), sharply decreased from 100 g, and the preference and satisfaction of 27 g smaller than this decreased sharply than 53 g.

This study is meaningful as it suggests a relationship regression model of confectionery box density, preference, and satisfaction in order to investigate the limit weight according to confectionery box size. The scatter plot of density, preference, and satisfaction of the 18 cookie boxes with the different sizes and weights showed the highest linear relationship when the density was logarithmically transformed. Note that preference and satisfaction increased to 0.37 g/cm^3^ density. Therefore, regression analysis using data with less than this density shows that the explanatory power of the regression model is very high (more than 90%). Therefore, we can set the weight by using the limit density of 0.37 g/cm^3^ of this study for a certain size in order to set the weight that increases the satisfaction and preference in the paper-boxed confectionery product. For example, a limit weight of 1000 cm^3^ volume is set to 370 g.

The preferred limit weights or densities suggested by this study are confined to confectionery products packed in paper boxes, which may vary depending on the product and the material. The preference for weight depends on the product [21], and this study was limited to cookies, the preferred product of weight. Since perceived weight is influenced by the material, this study limited the packing materials of confectionery products to paper. Affective studies based on weight and size on other products are required in order to determine whether marginal weight and density depend on the product, in which weight is a preferred characteristic. In addition, it is necessary to study how preference and satisfaction increase according to the weight reduction of the product, in which the lightness is preferred, and whether there is a preferred limit weight.

## 7. Conclusions

This study analyzed the preference and satisfaction of affective dimension according to the weight and size of confectionery box with perceived weight of sensory dimension. This study experimentally found that there is a weight limit that increases the preference and satisfaction of the confection box, unlike perceived weight, which has a positive correlation with the weight and size of the confection box. The present study analyzed the preference and satisfaction according to the density and suggested a limit density of 0.37 g/cm^3^ in order to simplify the preference and satisfaction limit weights according to the confectionery box size. The limit density suggested in this study could be used to determine the weight of cookie boxes.

## Figures and Tables

**Table 1:** Characteristics of participants’ body and snack usage.

Variable	Category	Count	% of Total
Gender	Male	16	30
	Female	38	70
BW ^a^ (kg)	BW< 60	20	37
	60 ≤ BW < 70	14	26
	70 ≤ BW < 80	12	22
	80 ≤ BW	8	15
Stature (cm)	Stature < 160	7	13
	170 ≤ Stature < 180	12	22
	170 ≤ Stature < 180	27	50
	180 ≤ Stature	8	15
FS ^b^	Snack	27	50
	Cookies	21	39
	Pie	3	6
	Other	3	6
EF ^c^	Almost nothing	17	31
	1–2 days/week	24	44
	3–5 days/weak	11	20
	Almost everyday	2	4
EA ^d^	Less than half	8	15
	More than half	22	41
	All	24	44
HC ^e^	Strongly disagree	15	28
	Disagree	29	54
	Neutral	6	11
	Agree and strongly agree	4	7

^a^ BW: Body weight, ^b^ FS: Favorite snack, ^c^ EF: Eating frequency, ^d^ EA: Eating amount, ^e^ HC: Health consider.

**Table 2:** Size and weight of cookie boxes.

Box Number	Weight (g)	Box Size
1	198	Small
2		Medium
3		Large
4	100	Small
5		Medium
6		Large
7	385	Small
8		Medium
9		Large

**Table 3:** Effects of size and weight of cookie box.

Factor	Statistics	Perceived Weight	Preference	Satisfaction
Weight	*F*(2, 106)	234.99	45.46	74.23
	*p*	<0.001	<0.001	<0.001
Size	*F*(2, 106)	82.02	33.68	56.52
	*p*	<0.001	<0.001	<0.001
Weight × Size	*F*(4, 212)	7.22	13.58	9.44
	*p*	<0.001	<0.001	<0.001

**Table 4:** Perceived weight comparison of the three cookie box sizes.

Weight (g)	Size (i)	Size (j)	Mean Difference (i–j)	SD	*p*
100	Small	Medium	15.46	2.95	<0.001
	Small	Large	32.76	4.03	<0.001
	Medium	Large	17.30	3.52	<0.001
198	Small	Medium	20.57	4.04	<0.001
	Small	Large	46.98	4.90	<0.001
	Medium	Large	26.41	3.39	<0.001
385	Small	Medium	30.67	7.28	<0.001
	Small	Large	71.39	10.42	<0.001
	Medium	Large	40.71	7.08	<0.001

**Table 5:** Perceived weight comparison of three cookie box weights.

Size	Weight (i)	Weight (j)	Mean Difference (i–j)	SD	*p*
Small	100	198	−58.63	4.68	<0.001
	100	385	−146.71	11.63	<0.001
	198	385	−88.08	9.04	<0.001
Medium	100	198	−53.52	3.21	<0.001
	100	385	−131.50	8.87	<0.001
	198	385	−77.98	7.65	<0.001
Large	100	198	−44.41	4.48	<0.001
	100	385	−108.08	6.39	<0.001
	198	385	−63.68	5.94	<0.001

**Table 6:** Preference comparison of the three cookie box sizes.

Weight (g)	Size (i)	Size (j)	Mean Difference (i–j)	SD	*p*
100	Small	Medium	1.99	0.38	<0.001
	Small	Large	3.83	0.40	<0.001
	Medium	Large	1.84	0.27	<0.001
198	Small	Medium	0.86	0.33	0.01
	Small	Large	2.67	0.54	<0.001
	Medium	Large	1.81	0.40	<0.001
385	Small	Medium	−0.21	0.23	0.37
	Small	Large	0.44	0.50	0.37
	Medium	Large	0.66	0.43	0.14

**Table 7:** Preference comparison of the three cookie box weights.

Size	Weight (i)	Weight (j)	Mean Difference (i–j)	SD	*p*
Small	100	198	−1.35	0.37	0.001
	100	385	−1.47	0.54	0.009
	198	385	−0.12	0.39	0.76
Medium	100	198	−2.46	0.43	<0.001
	100	385	−3.68	0.59	<0.001
	198	385	−1.19	0.40	0.004
Large	100	198	−2.52	0.33	<0.001
	100	385	−4.86	0.50	<0.001
	198	385	−2.34	0.42	<0.001

**Table 8:** Satisfaction comparison of the three cookie box sizes.

Weight (g)	Size (i)	Size (j)	Mean Difference (i–j)	SD	*p*
100	Small	Medium	2.11	0.37	<0.001
	Small	Large	3.82	0.40	<0.001
	Medium	Large	1.71	0.23	<0.001
198	Small	Medium	1.11	0.35	0.003
	Small	Large	3.41	0.54	<0.001
	Medium	Large	2.31	0.40	<0.001
385	Small	Medium	0.002	0.21	0.99
	Small	Large	1.24	0.46	0.009
	Medium	Large	1.24	0.43	0.005

**Table 9:** Satisfaction comparison of the three cookie box weights.

Size	Weight (i)	Weight (j)	Mean Difference (i–j)	SD	*p*
Small	100	198	−2.04	0.37	<0.001
	100	385	−2.46	0.50	<0.001
	198	385	−0.41	0.31	0.19
Medium	100	198	−3.05	0.42	<0.001
	100	385	−4.57	0.55	<0.001
	198	385	−1.52	0.44	0.001
Large	100	198	−2.45	0.33	<0.001
	100	385	−5.04	0.49	<0.001
	198	385	−2.59	0.45	<0.001

**Table 10:** Preference and satisfaction regression model with cookie box density.

	Adj-*R*^2^	Coefficient	*t*	*p*	Durbin Watson
Preference	0.92	6.06	9.09	<0.001	1.39
Satisfaction	0.95	7.01	10.98	<0.001	1.40

**Table 11:** Weight conditions for box sizes.

Size	Density (g/cm^3^)				
	0.05	0.10	0.19	0.37	0.72
Small (535.44 cm^3^)	27 g	53 g	100 g	198 g	385 g
Medium (1035.72 cm^3^)	53 g	100 g	198 g	385 g	755 g
Large (2067 cm^3^)	100 g	198 g	385 g	755 g	1480 g

Note that gray cells in Table 11 are included in the validation experiment, while white cells were included in the previous experiment.

**Table 12:** Preference and satisfaction regression model with cookie box density.

	Adj-*R*^2^	Coefficient	*t*	*p*	Durbin Watson
Preference	0.89	6.58	10.28	<0.001	2.33
Satisfaction	0.94	7.12	14.38	<0.001	1.89
